# Vector competence of selected North American *Anopheles* and *Culex* mosquitoes for Zika virus

**DOI:** 10.7717/peerj.4324

**Published:** 2018-02-15

**Authors:** Brittany L. Dodson, Sujit Pujhari, Jason L. Rasgon

**Affiliations:** The Department of Entomology, Center for Infectious Disease Dynamics, and the Huck Institutes of the Life Sciences, Pennsylvania State University, University Park, PA, USA

**Keywords:** Arbovirus, Vector-borne, Vector competence, Infection, Dissemination, Transmission

## Abstract

Zika virus (ZIKV) is a vector-borne flavivirus that has caused recent outbreaks associated with serious disease in infants and newborns in the Americas. *Aedes* mosquitoes are the primary vectors for ZIKV, but little is known about the diversity of mosquitoes that can transmit ZIKV in North America. We chose three abundant North American mosquito species (*Anopheles freeborni*, *Anopheles quadrimaculatus*, and *Culex tarsalis*) and one known vector species (*Aedes aegypti*), fed them blood meals supplemented with a recent outbreak ZIKV strain, and tested bodies, legs, and saliva for infectious ZIKV. ZIKV was able to infect, disseminate, and be transmitted by *Aedes aegypti*. However, *Anopheles freeborni*, *Anopheles quadrimaculatus*, and *Culex tarsalis* were unable to be infected. We conclude that these species are unlikely to be involved in ZIKV transmission in North America. However, we should continue to examine the ability for other mosquito species to potentially act as ZIKV vectors in North America.

## Introduction

Zika virus is an emerging mosquito-borne flavivirus that has caused epidemics in the Americas, Asia, Africa, and Pacific Islands (Roth et al., 2014; Guerbois et al., 2016; Hennessey, Fischer & Staples, 2016; Musso & Gubler, 2016; Tognarelli et al., 2016; Centers for Disease Control and Prevention, 2017a). According to the World Health Organization, at least 84 countries, territories, or subnational areas have evidence of ZIKV transmission, of which 61 were reintroductions and newly reported countries with no evidence of the virus before 2015 (World Health Organization, 2017). The virus was first identified in Uganda in 1947 and was restricted to epizootics in monkeys and sporadic infections in humans in Africa and Asia (MacNamara, 1954; Olson et al., 1981; McCrae & Kirya, 1982). ZIKV is primarily transmitted by mosquitoes, but has also been found in many human tissues and bodily excretions, and has the potential to be transmitted between humans through these fluids (Foy et al., 2011; Musso et al., 2014; Musso et al., 2015; Waddell & Greig, 2016; Atkinson et al., 2017). Symptomatic infections are usually self-limiting, causing rash and flu-like symptoms, but have also been associated with more severe complications including microcephaly in newborns and young children and Guillain–Barre syndrome in adults (Oehler et al., 2014; European Centre for Disease Prevention and Control, 2015; Mlakar et al., 2016; Rasmussen et al., 2016; Schuler-Faccini et al., 2016). There are no approved therapies or vaccines for ZIKV infection. To prevent infection, the suggestions are to avoid mosquito bites, control mosquito populations (e.g., source reduction, application of insecticides), and use condoms to prevent possible sexual transmission (Wong, Poon & Wong, 2016; Centers for Disease Control and Prevention, 2017b).

**Figure 1:**
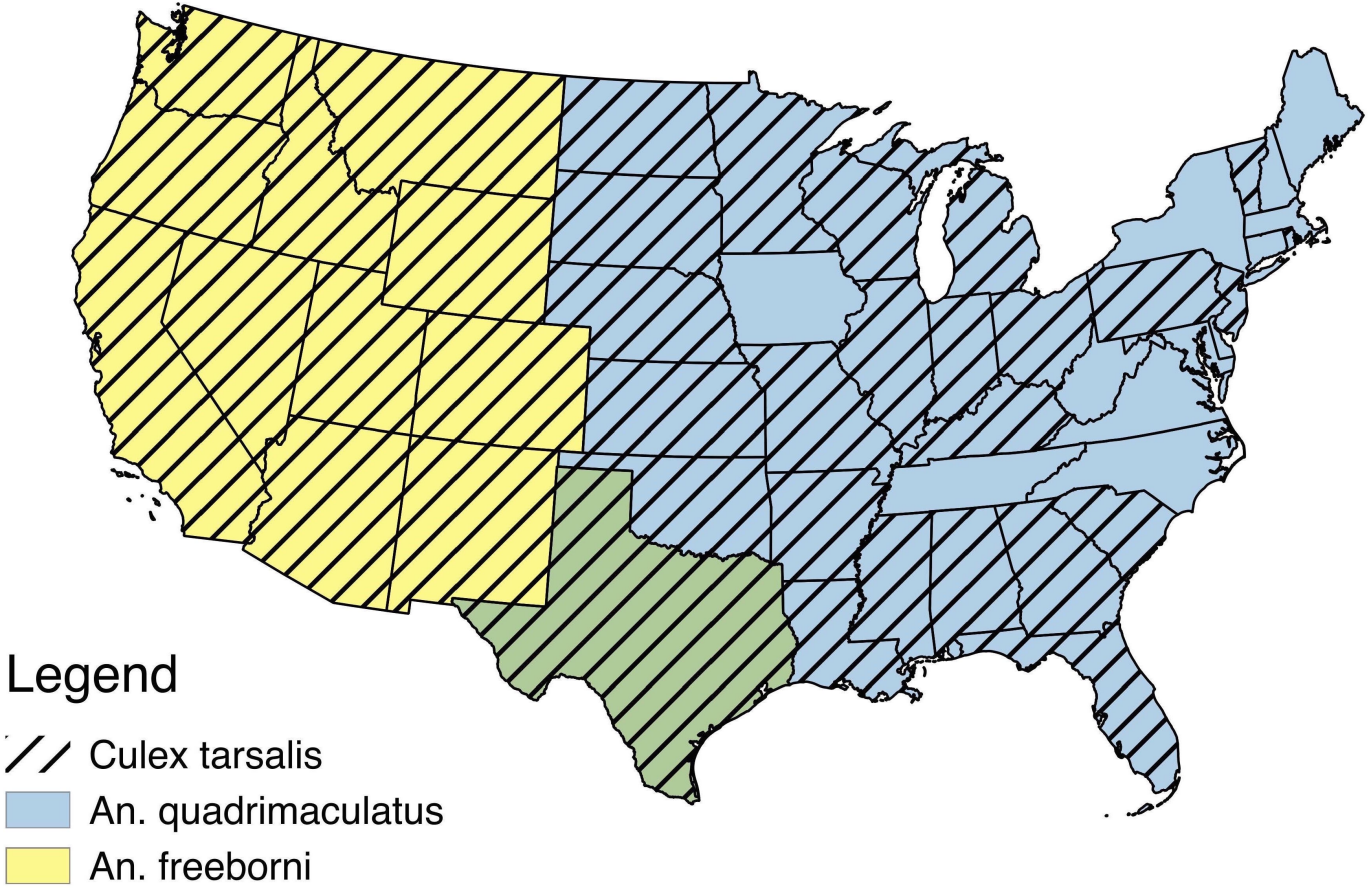
USA distribution by state of Anopheles freeborni, Anopheles quadrimaculatus, and Culex tarsalis (Hayes et al., 2005; Venkatesan & Rasgon, 2010; Manguin, 2013). An overlap of the distributions of *An. freeborni* and *An. quadrimaculatus* is denoted by the color green.

Despite a recent increase in the number of studies published about ZIKV, we still lack basic information about many aspects of its biology (Messina et al., 2016). In addition to an outbreak of locally acquired cases of ZIKV in Florida in 2016, the contiguous United States is predicted to be suitable for further ZIKV expansion based on a variety of environmental factors (Messina et al., 2016, “2016 case counts in the US,” 2017). We have little knowledge about what North American mosquitoes are capable of transmitting this virus, and although some *Anopheles* and *Culex* species have been shown to be inefficient vectors (Dodson & Rasgon, 2017; Kenney et al., 2017; Roundy et al., 2017) ZIKV has been isolated from these genera before (Pujhari & Rasgon, 2016). Primary mosquito vectors for ZIKV have been demonstrated to be in the *Aedes* genera, most notably *Ae. aegypti* and *Ae. albopictus* (Wong et al., 2013; Diallo et al., 2014; Grard et al., 2014; Gendernalik et al., 2017). Recent environmental suitability maps suggest that 77% of counties in the United States are suitable for both of these *Aedes* species, including the southeast, the south, southern portions of the northeast, and midwest, as well as coastal edges of the west (Johnson et al., 2017). ZIKV has been isolated from many other mosquito species and genera, but most of these have not been experimentally tested for their ability to serve as vectors (Faye et al., 2013; Waddell & Greig, 2016). For this study, we selected several mosquito species that are abundant across large areas of the United (*Anopheles freeborni*, *An. quadrimaculatus*, and *Culex. tarsalis* (Hayes et al., 2005; Venkatesan & Rasgon, 2010; Manguin , 2013) (Fig. 1), and evaluated their ability to become infected with and transmit ZIKV in the laboratory.

## Materials and Methods

### Mosquito and Zika virus propagation

Four mosquito species were tested for their ability to become infected with and transmit ZIKV. *Anopheles freeborni* (F1 strain, MRA-130), *An. quadrimaculatus* (Orlando strain, MRA-139), and *Culex tarsalis* (Yolo strain, NR-43026, colony originated from Yolo County CA in 2003) were provided by BEI resources (National Institute of Allergy and Infectious Diseases, Manassas, VA). *Aedes aegypti* (Rockefeller strain) were used as a positive control.

All mosquito species were reared and maintained at the Penn State Millennium Sciences Complex insectary at 27 °C ± 1 °C, 12:12 hr light:dark cycle, and 80% relative humidity in 30 × 30 × 30-cm cages. Mosquitoes were provided with cotton pads soaked with 10% sugar for maintenance and fed anonymous expired human blood (Biological Specialty Corporation, Colmar, PA; anticoagulant = citrate phosphate dextrose adenine) using a glass feeder jacketed with 37 °C distilled water for colony propagation and experimental virus infections. *Aedes aegypti* larvae were fed on whole koi pellets (TetraPond, Koi Vibrance, Prestons, Australia), all *Anopheles* larvae were fed finely ground koi pellets, and *Culex tarsalis* larvae were fed a 1:1:1 mixture by weight of ground bovine liver powder (MP Biomedicals, Solon, OH), koi pellets, and rabbit pellets (Kaytee, Chilton, WI, USA).

ZIKV strain PRVABC59 (provided by BEI Resources, NR-50240; GenBank KU501215) was used for all mosquito infections. This virus was originally isolated in 2015 from infected human serum in Puerto Rico and passed once in *Cercopithecus aethiops* kidney epithelial cells (Vero 76, ATCC CRL-1586). Working stocks were made by passing the virus three times in Vero cells (grown in Dulbecco’s modified-essential media (10566-024; Thermo Fischer Scientific, Gibco, Waltham, MA, USA) with 10% fetal bovine serum, and 1 × penicillin–streptomycin), harvesting the supernatant, and storing the 300 µl aliquots at −70 °C until used for mosquito infections.

### Mosquito vector competence for Zika virus

ZIKV aliquots were thawed briefly at 37 °C and were added to whole human blood in equal volumes. Aliquots were only thawed once to prevent any loss in virulence via multiple freeze–thaw cycles. We exposed adult female mosquitoes (3–7 days old) to ZIKV-infected blood via a glass feeder jacketed with 37 °C distilled water. Mosquitoes were allowed to feed to engorgement for up to 1 h. Mosquitoes were sorted by fed status under CO_2_ anesthesia. All mosquitoes that took a blood meal were fully engorged and they were retained for transmission experiments; non-blood fed females were discarded. We stored a 200–300 ml aliquot of each blood meal pre-feeding to confirm ZIKV titer by plaque assay on Vero cells, as described below.

We assessed ZIKV infection, dissemination, and transmission at 7 days post-blood feeding (Aitken, 1977; Dodson, Kramer & Rasgon, 2011; Dodson, Kramer & Rasgon, 2012). We attempted to assess additional time points, but (except for *Aedes aegypti*) feeding rates on the artificial blood meal were poor and resulted in low numbers; additionally, sufficient numbers of mosquitoes did not survive past 10 days post-feeding. Assayed female mosquitoes were anesthetized with triethylamine (Sigma, St. Louis, MO, USA). Legs from each mosquito were removed with forceps and placed in a 2-ml tube filled with 1 ml mosquito diluent (MD: 20% heat-inactivated fetal bovine serum [FBS] in Dulbecco’s phosphate-buffered saline, 50 µg/ml penicillin/streptomycin, 50 ug/mL gentamicin, and 2.5 µg/mL fungizone) with a single sterile, zinc-plated, steel 4.5 mm ball (Daisy, Rogers, AR). To induce mosquitoes to salivate, the mouthparts of each mosquito were placed in a tapered capillary tube containing approximately 10 µl of 1:1 50% sucrose and FBS (Moudy et al., 2007; Dodson, Kramer & Rasgon, 2011). Saliva was collected for 30 min, after which the contents were expelled into 0.3 ml MD. Bodies were placed individually into 2-ml tubes filled with 1 ml MD and a single sterile, zinc-plated, steel 4.5 mm BB. All samples were stored at −70 °C until tested for ZIKV by Vero plaque assay. All mosquito experiments with infectious ZIKV were conducted in the Eva J. Pell ABSL3 Laboratory at Pennsylvania State University, according to established biosafety protocols (IRB #45458).

### Zika virus plaque assay

We tested blood meals and mosquito samples for ZIKV infectious particles by Vero cell plaque assays (Payne et al., 2006). To identify infected mosquitoes, mosquito bodies were first assayed. If the body of that individual was positive, the legs and saliva were assayed to test for dissemination and transmission. Prior to the start of the assay, samples were thawed briefly at 37 °C. Bodies and legs were homogenized for 30 s with a TissueLyser (QIAGEN, Hilden, Germany) at 24 cycles/sec, and centrifuged for 1 min. Samples were kept on ice to prevent loss of viral infectivity during the assay, and after the initial infection concluded, samples were returned to −70 °C. Complete media (1 × Dulbecco’s modified-essential media (10566-024; Thermo Fischer Scientific, Waltham, MA, USA), 100 units/ml penicillin–streptomycin, and 10% fetal bovine serum) was removed from confluent monolayers of Vero cells in 6-well-plates, and monolayers inoculated with 100 µl of each clarified sample. Inoculated plates were placed in an incubator at 37 °C with 5% CO_2_ for 2 h and were rocked every 30 min to prevent cell desiccation. After the 2 h incubation, a 3 ml overlay was applied to each well that comprised equal amounts of complete media and 1.2% agarose, and plates incubated for 3 days. A secondary 3 ml overlay was then applied that comprised equal amounts of complete media and 1.2% agarose, as well as 1.5% neutral red solution, and plates incubated for one additional day. After incubation, plates were inverted, placed on a white light transilluminator (Hall Productions, San Luis Obispo, CA, USA), and each well assessed as positive or negative for ZIKV by observation of plaques. Plaques were counted if they were present. Positive and negative controls were included with every assay to confirm that the assay worked as expected and to ensure cell viability (positive: diluted ZIKV-infected blood meal and ZIKV stocks; negative: complete cell culture media and MD). Infection rate was defined as the proportion of mosquitoes exposed to virus that had ZIKV-positive bodies. Dissemination and transmission rates were defined as the proportion of mosquitoes infected with ZIKV that had infectious viral particles present in their legs and saliva, respectively.

## Results

ZIKV infectious particles were detected in all blood meals provided to mosquitoes and those blood meal titers were the following: experiment 1, 8.6 log10 plaque-forming units (PFU)/ml; experiment 2, 8.0 log_10_ PFU/ml; and experiment 3: 8.4 log_10_ PFU/ml. Additionally, we assayed several positive day 0 mosquitoes and detected infectious ZIKV, indicating that mosquitoes imbibed infectious blood. A summary of sample sizes and transmission assay results for each experiment are provided in Table 1. Despite feeding on a high ZIKV titer (≥8 logs), only the positive control species *Ae. aegypti* became infected with and transmitted ZIKV.

**Table 1:** Summary of Zika virus vector competence results.^a^^,^^b^

	Replicate 1				Replicate 2				Replicate 3				Total tested
Mosquito species	*N*	% I	% D^c^	% T^c^	*N*	% I	% D^c^	% T^b^^,^^c^	*N*	% I	% D^c^	% T^c^	–
*Ae. aegypti*	30	60	22	0	30	40	67	8	23	22	20	0	84
*An. quadrimaculatus*	19	0	0	0	11	0	0	0	4	0	0	0	34
*An. freeborni*	27	0	0	0	0	–	–	–	7	0	0	0	35
*Cx. tarsalis*	0	–	–	–	16	0	0	0	0	–	–	–	16

**Notes.**

a Blood meal titers for each replicate in order were as follows: 8.6 log_10_ PFU/ml, 8.0 log_10_ PFU/ml, 8.4 log_10_ PFU/ml.

b All mosquitoes were tested at seven days post blood feeding.

c Calculated based on number infected.

I Infected D Disseminated T Transmitted

## Discussion

In agreement with several other studies, the *Culex* and *Anopheles* species tested in this study were not competent for ZIKV infection (Weger-Lucarelli et al., 2016; Dodson & Rasgon, 2017; Kenney et al., 2017; Roundy et al., 2017). However, we cannot rule out the possibility that there are other members of these genera that could serve as vectors (Pujhari & Rasgon, 2016; Dodson & Rasgon, 2017). Additionally, while we chose an epidemiologically relevant outbreak ZIKV strain to assay, (PRVABC59) it is possible that there are differences in the permissibility of different ZIKV strains to infect mosquitoes (Moudy et al., 2007; Weger-Lucarelli et al., 2016). It is possible that if enough mosquitoes had bloodfed and survived to later time points, infection/transmission might have occurred. For example, one study reported that Rift Valley fever virus infection was 0% at day 7 and 50% at day 14 in *Culex pipiens* form molestus (Turell, Dohm & Fonseca, 2014). However, multiple studies show that 7 days post-infection is generally sufficient time for, at minimum, infection of the midgut epithelium to occur, and *Aedes aegypti* was able to become infected and even transmit at this time point (Goddard et al., 2002; Turell et al., 2008; Weger-Lucarelli et al., 2016; Moudy et al., 2007). Due to low colony size, and difficulty in getting mosquitoes to feed on an artificial blood meal, we only had enough *Cx. tarsalis* to conduct one infectious feed. However, our results are consistent with other work reporting that *Cx. tarsalis* appear not to be a competent ZIKV vector (Weger-Lucarelli et al., 2016); however, further studies should be conducted to confirm this finding.

## Conclusions

Our results suggest that *An. freeborni*, *An. quadrimaculatus*, and *Cx. tarsalis* are unable to become infected with ZIKV, and that these mosquito species are unlikely to contribute to transmission of this virus in North America.

## Additional Information and Declarations

### Data Availability

The following information was supplied regarding data availability: All raw data are reported in Table 1.
